# Synthesis and Design of Purpurin-18-Loaded Solid Lipid Nanoparticles for Improved Anticancer Efficiency of Photodynamic Therapy

**DOI:** 10.3390/pharmaceutics14051064

**Published:** 2022-05-15

**Authors:** Sooho Yeo, Hyeon Ho Song, Min Je Kim, Seokhyeon Hong, Il Yoon, Woo Kyoung Lee

**Affiliations:** Center for Nano Manufacturing, Department of Nanoscience and Engineering, Inje University, Gimhae 50834, Korea; thgugh@gmail.com (H.H.S.); minje520@hanmail.net (M.J.K.); d980321@naver.com (S.H.)

**Keywords:** photodynamic therapy, photosensitizers, purpurin-18, solid lipid nanoparticle, anti-cancer therapy

## Abstract

Purpurin-18 (P18) is one of the essential photosensitizers used in photodynamic therapy (PDT), but its hydrophobicity causes easy coalescence and poor bioavailability. This study aimed to synthesize P18 and design P18-loaded solid lipid nanoparticles (SLNs) to improve its bioavailability. The characteristics of the synthesized P18 and SLNs were evaluated by particle characteristics and release studies. The effects of P18 were evaluated using the 1,3-diphenylisobenzofuran (DPBF) assay as a nonbiological assay and a phototoxicity assay against HeLa and A549 cell lines as a biological assay. The mean particle size and zeta potential of the SLNs were 164.70–762.53 nm and −16.77–25.54 mV, respectively. These results indicate that P18-loaded SLNs are suitable for an enhanced permeability and retention effect as a passive targeting anti-cancer strategy. The formulations exhibited a burst and sustained release based on their stability. The DPBF assay indicated that the PDT effect of P18 improved when it was entrapped in the SLNs. The photocytotoxicity assay indicated that P18-loaded SLNs possessed light cytotoxicity but no dark cytotoxicity. In addition, the PDT activity of the formulations was cell type- and size-dependent. These results suggest that the designed P18-loaded SLNs are a promising tool for anticancer treatment using PDT.

## 1. Introduction

Photodynamic therapy (PDT) is an innovative and attractive cancer treatment strategy [1]. PDT is patient-friendly, as it selectively destroys tumor cells through the interaction with a photosensitizer (PS) in the presence of light [1,2]. PS exerts its pharmacological effect through photoirradiation, which generates reactive oxygen species, especially singlet oxygen (^1^O_2_) [1,2,3]. The generated ^1^O_2_ triggers cell death via oxidative damage, which leads to the apoptosis of cancer cells without affecting the adjacent healthy cells [4].

PDT researchers continue to search for PSs that interact with relatively long wavelengths because light with long wavelengths easily penetrates the human body, leading to effective treatment of deeply placed tumors [5,6]. Purpurin-18 (P18) is a chlorin class PS synthesized from chlorophyll [6]. The molecular weight of P18 is 564.6 g/mol, its chemical formula is C_33_H_32_N_4_O_5_, and its chemical name is (15S,16S)-10-ethenyl-5-ethyl-16,18-dihydro-6,11,15,22-tetramethyl-18,20-dioxo-15H,20H-4,7:14,17-diimino-2,21-metheno-9,12-nitrilopyrano-[4,3-b]azacyclononadecine-16-propanoic acid [7,8,9]. P18 contains a fused anhydride and an aliphatic side chain terminated with a carboxylic group, resulting in its interaction with a relatively long wavelength of more than 650 nm [5,6]. Thus, deeply penetrating light can be used for PDT using P18. However, the hydrophobicity of P18 leads to coalescence at physiological pH, reducing its bioavailability and pharmacological effect [1,6,7,9].

Various approaches have been reported to improve the physicochemical characteristics of P18, such as core metalation [10], PEGylation [11,12,13,14], and conjugation with peptides [15,16,17,18], choline [19,20], and gold nanoparticles [19]. However, considering safety issues related to core metalation and gold nanoparticles, as well as the storage stability of formulations associated with water-soluble polymers, pharmaceutical technologies for improving the bioavailability of PS need to be investigated [1,21]. In cancer therapy, one of the traditional pharmaceutical strategies is passive targeting through the enhanced permeability and retention (EPR) effect [21,22]. Nanocarriers up to a size of 400 nm allow PS accumulation at the tumor site [23].

Lipid-based drug delivery systems, including microemulsions, lipid nanocapsules, self-emulsifying systems, and liposomes, have been widely used to increase the aqueous solubility and stability of drugs, to improve their bioavailability [20,21,24,25]. Despite these promising pharmaceutical strategies, formulations still have issues such as low drug-loading capacity, usage of bio-toxic organic solvents, and instability in biological fluids [3,26,27,28]. The solid lipid nanoparticle (SLN) is a promising formulation among lipid-based drug delivery systems because of its relatively high drug-loading capacity, organic-solvent-free formulation, and structural separation of the drug from the external environment [29,30,31]. In SLNs, a drug is entrapped in a lipid matrix via the preparation of an oil-in-water (O/W) phase without using organic solvents, which leads to a relatively high drug-loading capacity and substantially reduced risk of bio-toxicity [30,31,32]. Interestingly, the drugs entrapped in SLNs are easily delivered to the lymphatic circulation via chylomicron formation in enterocytes, which results in bypassing of the liver [26]. Hence, SLN systems effectively inhibit the degradation of entrapped drugs by the hepatic first-pass metabolism [27].

The present study aimed to synthesize P18 from chlorophyll and design P18-loaded SLNs to improve its bioavailability and anticancer effects in PDT. The structure and absorption wavelength of the synthesized P18 were assessed using ^1^H nuclear magnetic resonance (^1^H-NMR) and UV-Vis spectroscopy, respectively. The pharmaceutical properties of the P18-loaded SLNs were evaluated by analyzing their particle size, zeta potential, and photostability, and the chemical interactions between P18 and the SLN ingredients were evaluated by Fourier transform infrared (FTIR) spectroscopy. The pharmacological effect of PDT using P18-loaded SLNs was determined by measuring ^1^O_2_ photogeneration using the 1,3-diphenylisobenzofuran (DPBF) nonbiological assay and the in vitro phototoxicity against two tumor cell lines (HeLa and A549) using the WST biological assay under dark and light conditions using LED light that can irradiate a wide range of wavelengths.

## 2. Materials and Methods

### 2.1. Materials

Phosphate-buffered saline (PBS), methylene blue (MB), and chloroform were purchased from Sigma-Aldrich (St. Louis, MO, USA). Lauric acid (LA) and palmitic acid (PA) were purchased from SAMCHUN (Pyeongtaek, Korea). Glycerol monostearate (GMS) was purchased from Kanto Chemical Co, Japan. Inc. (Tokyo, Japan). Tween 80 (TW 80) was purchased from Dae Jung Co., Ltd. (Busan, Korea). Chlorophyll-a paste was purchased from Shandong Lanmo Biotech Co. Ltd. (Shanghai, China). Methylene chloride (CH_2_Cl_2_, MC) was purchased from Duksan Co. Ltd. (Ansan-si, Gyeonggi-do, Korea). DPBF was purchased from TCI Chemicals (Tokyo, Japan). Dulbecco’s modified Eagle’s medium (DMEM) was purchased from WelGENE (Gyeongsan, Korea). Penicillin-streptomycin solution (100×) and fetal bovine serum (FBS) were purchased from BioWest (Nuaillé, France). The cancer cell lines (HeLa and A549) were obtained from the Korean Cell Line Bank (Seoul, Korea). The Quanti-MAX WST-8 assay kit was purchased from Biomax (Seoul, Korea). High-performance liquid chromatography (HPLC)-grade methanol (MeOH) was purchased from Honeywell (Seelze, Germany). All other chemicals used were of HPLC grade.

### 2.2. Synthesis of P18

Methyl pheophorbide-a (MPa) was extracted through acidic treatment of chlorophyll-a paste according to a previously reported protocol [33]. Briefly, MPa (1 g) was added to pyridine (5 mL), diethyl ether (400 mL), and KOH solution (12 g KOH dissolved in 80 mL of 1-propanol), and stirred under aeration for 3 h. The resulting solution was separated by pouring distilled water (DW). The organic layer thus obtained was evaporated. To obtain pure P18, the residue was separated by column chromatography using 5% MeOH/MC as the eluent.

### 2.3. Preparation of P18-Loaded SLNs

P18-loaded SLNs with various compositions were prepared using a modified O/W emulsion method. Briefly, P18 was dissolved in melted lipid at 10 °C above the lipid melting point, and homogenized using a polytron homogenizer (PT 3100; Kinematica instruments, Luzerne, Switzerland) at 1000 rpm, which led to an oil (O) phase. To produce the O/W emulsion, the O phase and the outer aqueous (W) phase containing TW 80 were mixed using a polytron homogenizer. SLNs were then prepared using a probe sonicator (Scientz-IID, Ningbo, China) at 300 W for 15 min, with a 5 s pulse-on period and a 5 s pulse-off period. The different compositions of P18-loaded SLNs are shown in Table 1.

### 2.4. Characterization of P18

#### 2.4.1. NMR Spectroscopy

The synthesized P18 was characterized by ^1^H-NMR using a Varian spectrometer (500 MHz) at the Biohealth Products Research Center of Inje University, Republic of Korea. For preprocessing, the specimen was dissolved in CDCl_3_.

#### 2.4.2. Development of Analytical Method for P18

The analysis of P18 was used by a UV-Vis spectrophotometer (S-3100; Scinco, Seoul, Korea) at ambient temperature. The wavelength of P18 was determined in the range of 300–800 nm. A standard stock solution and each sample were dissolved in MeOH at the concentration of 2 mg of P18 in 20 mL.

##### Linearity

To determine calibration curves, the five concentrations (1–20 ppm) of standard stock solutions were prepared via dilution in MeOH. P18 in each formulation constructed calibration curves and concentration versus absorbance units.

##### Precision and Accuracy

The precision was determined by analyzing the six replicate determinations of stock solution with one concentration. The accuracy was prepared by the recovery of stock solutions with 0%, 25%, and 100% levels. The precision and accuracy were expressed as the relative standard deviation (RSD) by calculating the recovery of the stock solutions.

### 2.5. Characterization of P18-Loaded Nanotransformers

#### 2.5.1. Determination of Particle Characteristics

The particle characteristics (nanoparticle size, polydispersity index (PDI), and zeta potential) were determined by dynamic light scattering using a Zetasizer Nano ZS (Malvern Instruments Ltd., Worcestershire, Malvern, UK). To make the pretreatment, all samples were diluted 10 times with DW, respectively. Each value reported is the triplicate average of measurements.

#### 2.5.2. Determination of Drug-Loading Capacity

The loading efficiency (LE) and loading amount (LA) of P18 in P18-loaded SLNs were analyzed by a UV-Vis spectrophotometer. The diluted SLNs were centrifuged at 1300 rpm at 4 °C for 1 h. The concentration of free drug in the supernatant was estimated by a UV-Vis spectrophotometer as described in Section 2.4.2. To obtain loading capacity, the estimated P18 was calculated by using Equations (1) and (2), respectively:

(1) $$\mathrm{LE}\;\left(\%\right)=\frac{\mathrm{Amount}\;\mathrm{of}\;\mathrm{total}\;\mathrm{drug}\;\mathrm{content}-\mathrm{Amount}\;\mathrm{of}\;\mathrm{free}\;\mathrm{drug}}{\mathrm{Amount}\;\mathrm{of}\;\mathrm{total}\;\mathrm{drug}\;\mathrm{content}}\times 100$$

(2) $$\mathrm{LA}\;\left(\%\right)=\frac{\mathrm{Amount}\;\mathrm{of}\;\mathrm{total}\;\mathrm{drug}\;\mathrm{content}-\mathrm{Amount}\;\mathrm{of}\;\mathrm{free}\;\mathrm{drug}}{\left(\mathrm{Amount}\;\mathrm{of}\;\mathrm{total}\;\mathrm{drug}\;\mathrm{content}-\mathrm{Amount}\;\mathrm{of}\;\mathrm{free}\;\mathrm{drug}\right)+\mathrm{Amount}\;\mathrm{of}\;\mathrm{lipid}}\times 100$$

#### 2.5.3. FTIR-Attenuated Total Reflection (ATR) Spectroscopy

The FTIR study for P18 and the main ingredients of SLN was performed by an FTIR-ATR spectrometer (Spectrum Two FT-IR Spectrometer, PerkinElmer, Norwalk, CT, USA) equipped with a ZnSe crystal at the wavenumber range of 4000–800 cm^−1^.

### 2.6. In Vitro P18-Release Studies

An in vitro P18 release study from P18-loaded SLNs was carried out using the dialysis-bag method. Each test substance was added in dialysis bags (Spectrum Laboratories, Inc., Compton, CA, USA) with a molecular weight of 10 kDa, followed by immersing them in 50 mL of receptor medium (PBS, pH 7.4). The vials containing receptor medium were shaken using a shaking incubator (JSSI-100T, JS Research Inc., Gongju, Korea) at 100 rpm and 37 ± 0.5 °C. Aliquots of 1 mL were withdrawn from the receptor medium at predetermined time intervals (1, 2, 4, 8, 12, 24, and 48 h) and immediately analyzed using a UV-Vis spectrophotometer, as described in Section 2.4.2.

### 2.7. Photostability Studies

The photostability of P18 in SLN was carried out by the modified method described by Lima et al. [34]. Briefly, test substances were dissolved in a 0.1% MeOH solution at the concentration of 4.0 ppm. The dissolved samples were then irradiated with LED. At predetermined time intervals (0, 10, 20, 30, and 40 min), aliquots of 1 mL were collected from the vials, followed by adding 1 mL of hexane. A 0.1% MeOH layer was measured using a UV-Vis spectrophotometer as described in Section 2.4.2.

### 2.8. ^1^O_2_ Photogeneration

An amount of 1 μM of each sample with 50 μM of DPBF in DMSO was used to determine ^1^O_2_ photogeneration. Amounts of 50 μM DPBF and 1 μM MB with 50 μM DPBF were subjected to the negative control (NC) and positive control (PC), respectively. The prepared samples in a 48-well plate were irradiated (2 J/cm^2^) with an LED for 15 min. The absorbance of each sample was measured at 418 nm using a microplate reader (Synergy HTX; BioTek, Winooski, VT, USA).

### 2.9. In Vitro Photoirradiation Studies

#### 2.9.1. Cytotoxicity Study Using Human Tumor Cell Lines

The photocytotoxicity for P18-loaded SLNs was carried out using two tumor cell lines (HeLa from human cervical carcinoma and A549 from human lung carcinoma). A quantity of 2 × 10^4^ cells/well of calculated cells were seeded into 48-well plates, followed by incubation for 24 h at 37 ± 0.5 °C in a humidified atmosphere with 5% CO_2_. After incubation, 1, 2.5, 5, and 10 μM of each sample were added to each well. After administration for 24 h, the cells were rinsed by sterile PBS. The growth medium (200 µL/well) was added, followed by irradiation (2 J/cm^2^) with an LED at a distance of 20 cm for 15 min. To conduct the WST reduction assay, the treated cells were incubated for 24 h at 37 ± 0.5 °C and 5% CO_2_.

#### 2.9.2. Viability of Cancer Cells

The viability of cells was estimated according to the modified method described by Alépée [35]. Briefly, the incubated cells in 48-well plates were applied to 10% WST solution for 1 h. The concentration of WST was estimated using a microplate reader at 450 nm via determination of the optical density (OD). After subtracting the blank OD from all raw data, the triplicate experiments were represented the mean OD values ± standard deviations (SDs). The percentage of viability relative to that of the NC was calculated using Equation (3). The NC value was set at 100%.

(3) $$\mathrm{Viability}\;\left(\%\right)=\frac{{\mathrm{Mean}\;\mathrm{OD}}_{\mathrm{treated}}}{{\mathrm{Mean}\;\mathrm{OD}}_{\mathrm{control}}}\times 100$$

### 2.10. Cellular Accumulation Study

Two tumor cell lines (HeLa and A549) were seeded into confocal dishes at 2 × 10^4^ cells/dish followed by a 24 h post-incubation. The incubated cells were exposed to the high anti-cancer effect formulation (F3) at 10 μM corresponding to P18 for 24 h. The exposed cells were then fixed using paraformaldehyde, followed by staining by applying diamidino-2-phenylindole (DAPI) to the fixed cells. Cellular uptake images were observed under a confocal laser scanning microscope (CLSM, LSM 510 META, Carl Zeiss, Oberkochen, Germany) at Inje University.

### 2.11. Statistical Analysis

Three independent experiments were performed for all analyses. The presented data (mean ± SD) were compared using one-way analysis of variance and Student’s *t*-tests. Statistical significance was set at *p* < 0.05.

## 3. Results and Discussion

### 3.1. Characterization of P18

#### 3.1.1. NMR Spectroscopy

**Figure 1:**
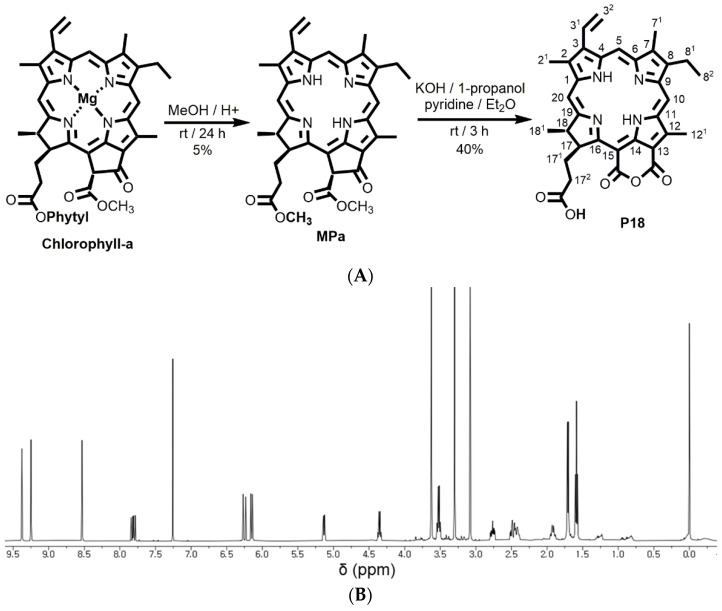
(**A**) Schematic representation of the synthesis of P18 from chlorophyll-a through MPa with numbering, (**B**) ^1^H-NMR spectrum of P18 (500 MHz, CDCl_3_, 25 °C, TMS).

The structure of P18 was characterized by ^1^H-NMR spectroscopy. Figure 1 shows the ^1^H-NMR spectrum of P18. ^1^H-NMR (500 MHz, CDCl_3_, 25 °C, TMS): δ 9.38 (s, 1H, 10H), 9.25 (s, 1H, 5H), 8.53 (s, 1H, 20H), 7.84 (dd, *J* = 17.8, 11.5 Hz, 1H, 3^1^H), 6.27 (d, *J* = 17.9 Hz, 1H, 3^2^H), 6.16 (d, *J* = 11.5 Hz, 1H, 3^2^H), 5.14 (m, 1H, 17H), 4.36 (m, 1H, 18H), 3.63 (s, 3H, 12^1^H), 3.53 (q, *J* = 7.7 Hz, 2H, 8^1^H), 3.30 (s, 3H, 2^1^H), 3.08 (s, 3H, 7^1^H), 2.76 (m, 1H, 17^2^H), 2.52–2.39 (m, 2H, 17^1^H), 1.93 (m, 1H, 17^2^H), 1.72 (d, *J* = 7.3 Hz, 3H, 18^1^H), 1.59 (t, *J* = 7.7 Hz, 3H, 8^2^H), and 0.01 and −0.23 (all brs and each 1H, NH). Our results indicate that the peaks for the two OCH_3_ groups disappeared from the MPa structure owing to the formation of a six-membered purpurin-18 ring.

#### 3.1.2. Development of Analytical Method for P18

##### The Absorption Spectra and Specificity of P18

**Figure 2:**
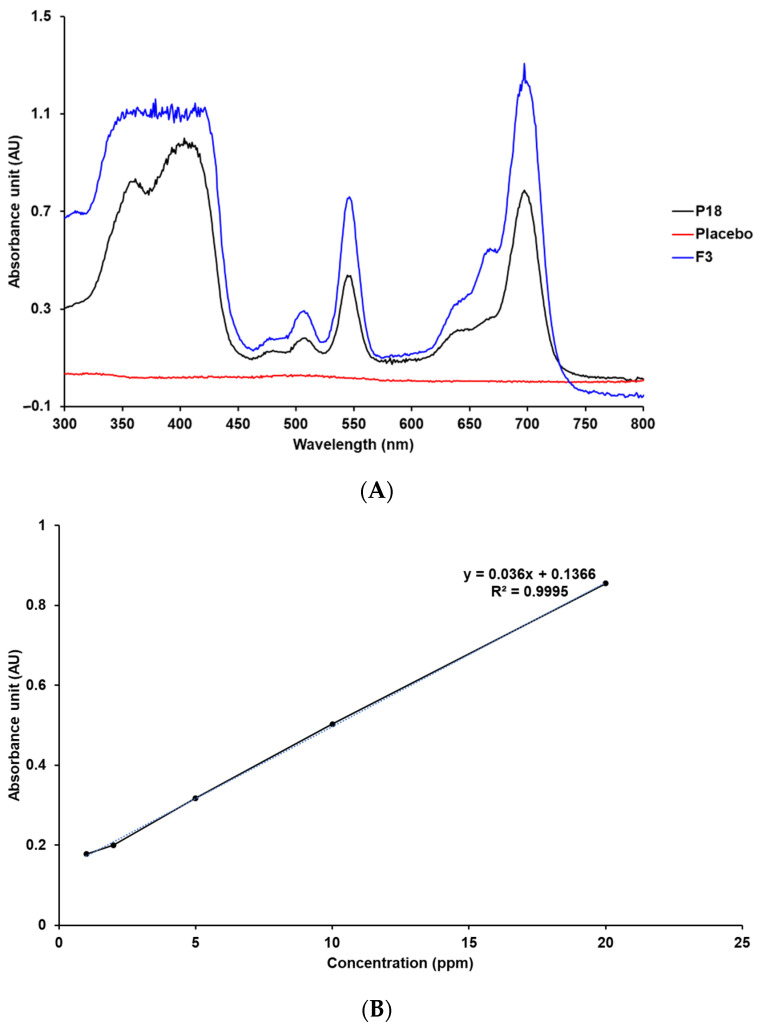
UV-Vis spectra and calibration curve of P18. (**A**) Specificity data of P18, placebo, and P18-loaded SLN F3 (MeOH, 25 °C). (**B**) Linearity data of P18 standard stock solution in MeOH.

The absorption spectra and specificity of P18 were estimated by determining both the maximum absorption wavelength and the specific absorption spectra [36,37,38]. The UV-Vis spectrum of P18 indicated that the maximum absorption wavelength was 700 nm, as shown in Figure 2. The UV-Vis spectra for the placebo SLN that was the non-P18 SLN in the same composition of F3 showed that there was no interference of the placebo with the analyte. Therefore, P18 was detected at a wavelength of 700 nm.

##### Linearity

Five standard stock solutions with P18 concentrations in the range of 1–20 ppm were analyzed for preparing a calibration curve. The correlation coefficient of the calibration curve was determined to be 0.9995 using linear regression analysis, as shown in Figure 2.

##### Precision

Precision, which describes the closeness of agreement among measurements for the same concentration of the standard stock solution, was indicated as the RSD of repeatability. The RSD (%) values of the recovery were determined to be 0.49%, as shown in Table 2. This indicated the high precision of the proposed analytical method.

##### Accuracy

Accuracy, which indicates the closeness of agreement between the test results and a conventional true or accepted reference value, was also expressed as the RSD calculated from the drug recovery. The RSD (%) values of the recovery were determined to be 1.52%, 0.52%, and 0.14%, respectively, as shown in Table 3. This indicates the high accuracy of the analytical method developed.

### 3.2. Characterization of P18-Loaded SLNs

#### 3.2.1. Nanoparticle Size, PDI, and Zeta Potential

**Figure 3:**
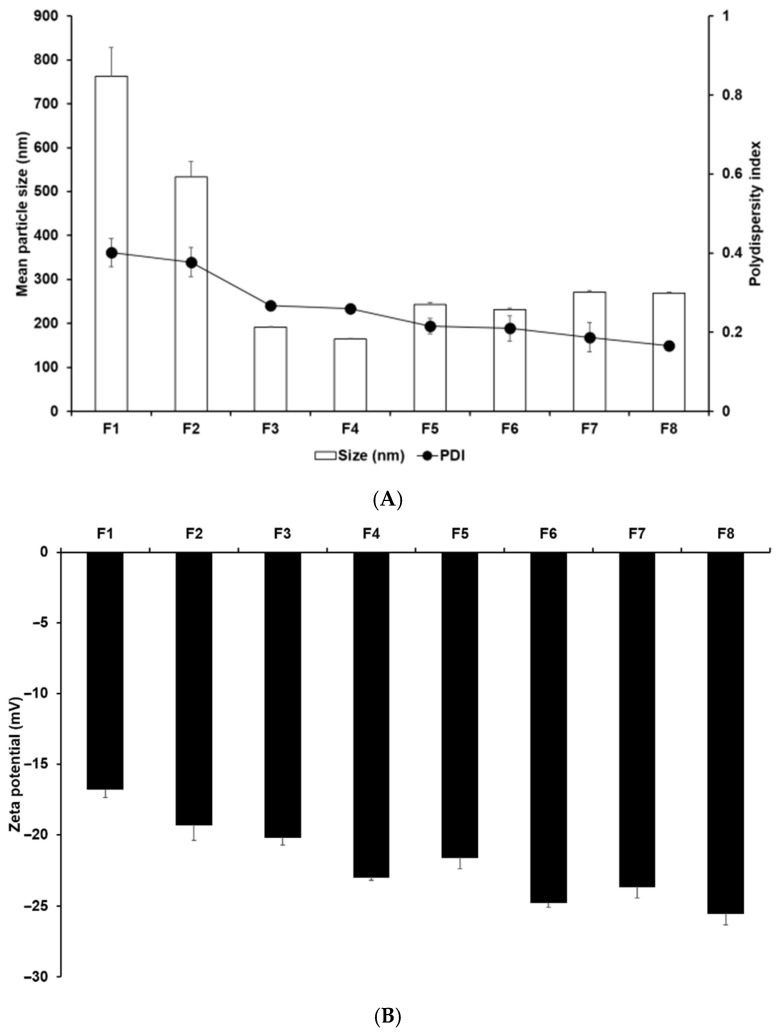
(**A**) Average particle sizes and PDIs and (**B**) zeta potentials of P18-loaded SLNs prepared using different ingredients. Results are expressed as the means ± standard deviations of three independent experiments (*n* = 3). PDI, polydispersity index.

Particle characterization studies were conducted to determine the effects of lipids and the concentrations of both lipids and surfactants on the EPR effect. Particle size is important for the EPR effect [3,26,39]. The zeta potential of nanoparticles represents their electric surface potential, and reflects their particle stability [26,27]. Nanoparticles with a high zeta potential prevent particle coalescence by repulsing each other, which ensures high storage stability and delayed drug release [40]. Thus, it is important to adjust the zeta potential to balance the drug release and storage stability of the formulation. The analysis of particle size, PDI, and zeta potential indicated that among F1–F3, which were formulations containing different lipids, F3 SLNs were the smallest, most homogeneous, and most stable, as shown in Figure 3. Particularly, the sizes of F1, F2, and F3 SLNs were 762.53 nm, 533.83 nm, and 191.03 nm, respectively. Thus, F3 appears to be a suitable formulation for application in the EPR effect-based PDT. The analysis of the effect of different lipids suggested that the formulations using longer-carbon-chain lipids had a higher affinity for P18 because high affinity between the drug and the lipids leads to easy dissolution of the drug into the lipids when producing the O phase of SLNs [24,25,40]. Hence, the drugs molecularly dispersed into the lipids of the O phase do not interfere with the energy of sonication that is used to produce the SLNs. The negative charge of the zeta potential was mainly attributed to the carboxyl group of the lipids because the surfactant was nonionic [41].

Regarding the effects of lipid and surfactant concentrations for F3–F8, the results indicated that an increase in the concentration of GMS as a lipid increased the particle size and zeta potential, as shown in Figure 3. An increase in the concentration of TW 80 as a surfactant decreased the particle size and increased the zeta potential. This suggests that an increase in the concentration of lipids causes an increase in the volume of the lipid matrix [42]. TW 80, a hydrophilic surfactant, stabilized the interface between O and W phases. This indicates that an increase in the concentration of TW 80 amplifies the stabilization effect at the interface between the O and W phases, which causes the particles to be smaller and more stable.

#### 3.2.2. Determination of the Drug-Loading Capacity

**Figure 4:**
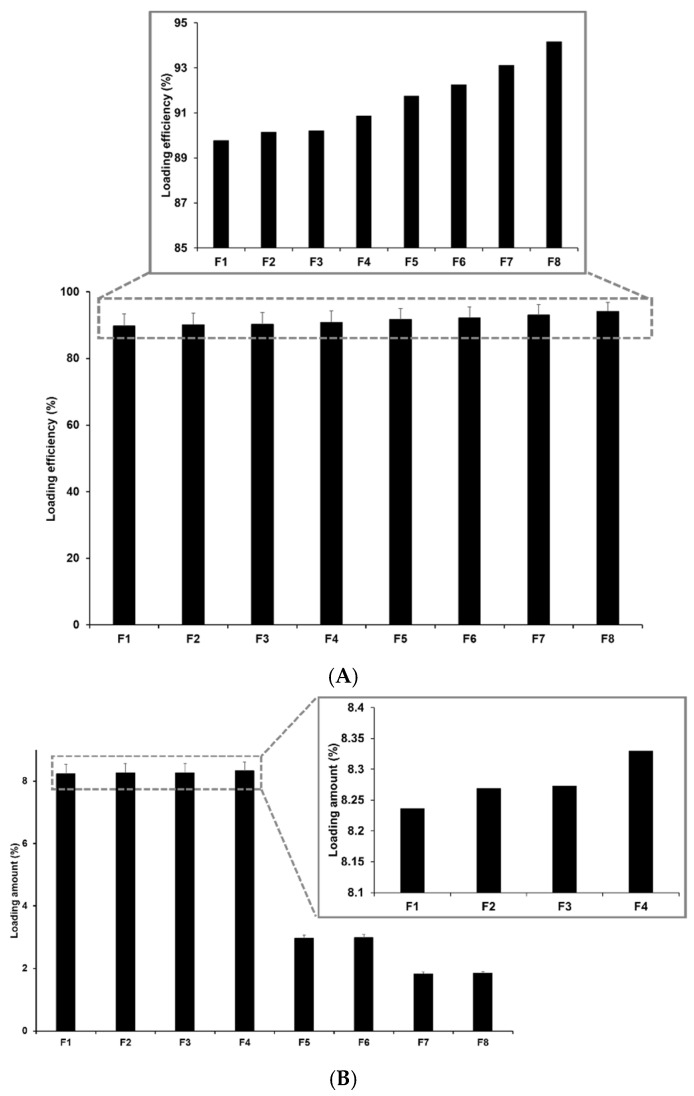
(**A**) Loading efficiency and (**B**) loading amount of P18-loaded SLNs prepared using different compositions. Results are expressed as the means ± standard deviations of three independent experiments (*n* = 3).

The drug-loading capacity plays an important role in drug delivery systems because it avoids side-effects and degradation and enables sustained release. The loading capacity (LE and LA) for P18-loaded SLNs ranged from 89.77% to 94.16% for LE and 1.83% to 8.33% for LA, as shown in Figure 4. Among the formulations using different lipids, the loading capacity of P18 in the SLNs increased slightly with an increase in the length of the lipid carbon chain. This suggests that the high affinity between P18 and lipids increases the solubility of P18 in the lipid matrix, as suggested by previous studies on particle size and zeta potential [24,25,40]. Regarding the concentrations of lipids and surfactants, an increase in the concentration of these ingredients increased the loading capacity of P18 in SLNs. This was because of the increase in the volume of the lipid matrix and the stabilized interface between the O and W phases, as mentioned in the previous studies on size and zeta potential [28,40,41]. In addition, the effect of the concentrations of lipids and surfactants on the loading capacity indicated that the effect of lipids was stronger than that of surfactants.

#### 3.2.3. FTIR-ATR Spectroscopy

**Figure 5:**
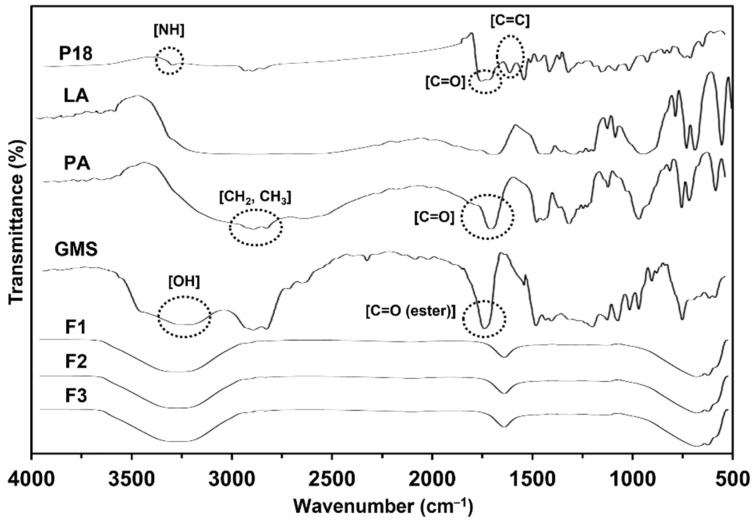
FTIR-ATR spectroscopy overlay spectra of solid lipid nanoparticles. Pure P18; LA; PA; GMS; F1: SLNs prepared using LA; F2: SLNs prepared using PA; F3: SLNs prepared using GMS. P18, purpurin-18; LA, lauric acid; PA, palmitic acid; GMS, glycerol monostearate.

The chemical interactions between P18 and the ingredients of the SLN were studied using FTIR-ATR spectroscopy. F1, F2, and F3 were selected as test substances because they were the main components used in the formulations. The FTIR spectrum of the synthesized P18 exhibited characteristic peaks at 1603 cm^−1^ (C=C stretching), 1714 cm^−1^ (C=O stretching), and 3334 cm^−1^ (N–H stretching), as shown in Figure 5 and summarized in Table 4. The FTIR spectra of the lipids (LA, PA, and GMS) also showed characteristic peaks at 3244 cm^−1^ (OH stretching in GMS), 2851 and 2919 cm^−1^ (CH_2_, CH_3_ stretching), 1702 cm^−1^ (C=O stretching in LA and PA), 1734 cm^−1^ (C=O (ester) stretching in GMS), and 1465 cm^−1^ (COOH stretching in LA and PA). The FTIR spectra of F1, F2, and F3 showed that there was a shift to 3304 cm^−1^ (OH stretching) and 1638 cm^−1^ (C=O stretching), the peaks for N–H stretching and C=C stretching were no longer detected in P18, and those for CH_2_, CH_3_ stretching, C=O (ester) stretching, and OCO stretching were no longer detected in lipids. This indicates hydrogen bond (H-bond) and van der Waals interactions between P18 and lipids [7,8,9]. The H-bond interactions of P18 with lipids occurred between N–H stretching and C=O stretching of P18; and C=O stretching and COOH stretching of LA and PA, or OH stretching and C=O (ester) stretching of GMS. The van der Waals interactions occurred between the C=C stretching of P18 and CH_2_ and CH_3_ stretching of lipids. Considering that the peaks for C=O and N–H of P18 were slightly shifted and not detected, respectively, the strong H-bonding between P18 and the lipids might be attributed to the N–H site of P18. Regarding the H-bonding between P18 and GMS, the significantly different structure of GMS compared with those of LA and PA was because of glycerol being its precursor [32]. Thus, the OH site in the GMS contributed to H-bonding, as indicated by the peak shift. In this regard, the particle size could also be relevant as the relatively high affinity of P18 for GMS is attributed to the supplementary H-bond (OH in GMS).

### 3.3. In Vitro P18-Release Analysis

**Figure 6:**
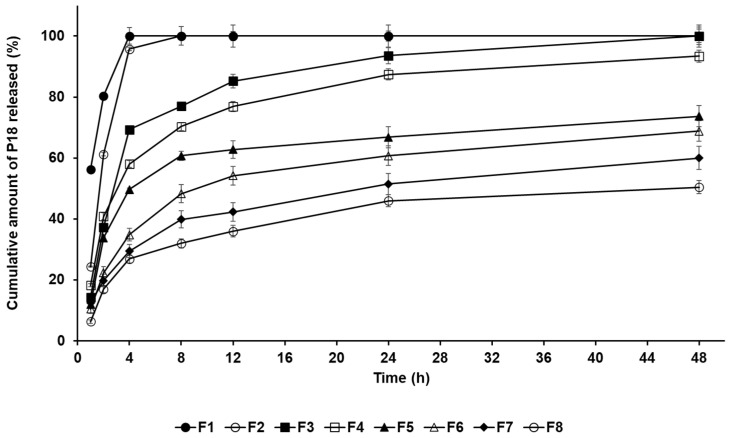
Cumulative percentage release profiles of P18 from SLNs F1–F8 in release medium, as determined using dialysis method. Results are expressed as the means ± standard deviations of three independent experiments (*n* = 3).

P18 release from the SLNs was studied using dialysis. Figure 6 shows the P18 release from F1 to F8. The results for F1, F2, and F3, which contained LA, PA, and GMS, respectively, indicated that F1 and F2 performed a burst release, whereas F3 performed a burst release over 4 h, followed by a sustained release until 48 h. This suggests that lipids with a higher affinity for P18 inhibited its release from P18-loaded SLNs, resulting in a sustained release of P18 [28]. The results for F3 to F8 indicated that an increase in the amount of either lipid or surfactant gradually delayed the P18 release from those formulations. The reason for this was that the volume of the lipid as a drug-entrapping matrix increased, which increased the drug-loading capacity and delayed the release of the entrapped drug [28,40,41]. Regarding the effects of the surfactant, the improvement in the stability of the interface between O and W phases increased the drug-loading capacity of the SLNs. Thus, the P18 release from the formulation was delayed [28]. Interestingly, the release profiles of F3–F8 exhibited a biphasic form, characterized by a relative burst release over 4 h, followed by a sustained release until 48 h. This suggests that the relative burst release of P18 was because of adhesion to the particle surface, while the sustained release was because of the entrapped drug inside the particles [28,39].

### 3.4. Photostability Studies

**Figure 7:**
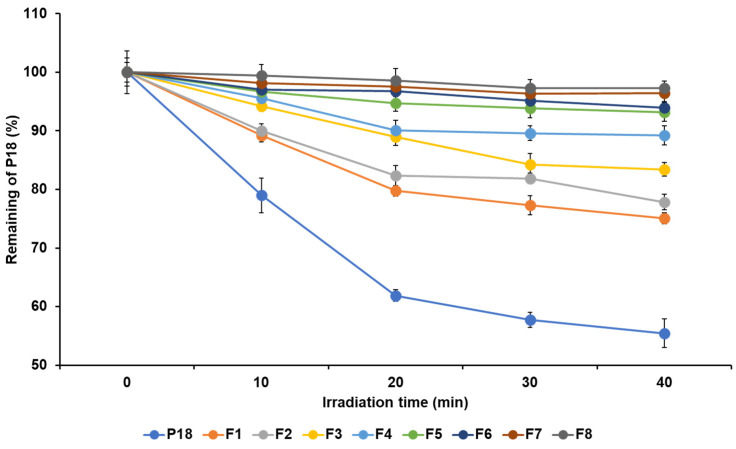
Photostability test based on percentage of nondegraded P18 in pure P18 solution and P18-loaded SLNs before and after irradiation of 2 J/cm^2^ with LED, for time intervals of 0, 10, 20, 30, and 40 min. Results are expressed as means ± standard deviations of three independent experiments (*n* = 3).

The photostability of P18 is important in light-induced therapies, including PDT. The photostability analysis indicated that all formulations enhanced the stability of P18 by light, as shown in Figure 7. After photo-irradiation for 40 min, the percentage of P18 remaining in the P18 solution and P18-loaded SLNs was 55.45% and ranged from 75.07% to 97.29%, respectively. The order of photostability was F8 > F7 > F6 > F5 > F4 > F3 > F2 > F1 > pure P18. This suggests that the SLN structurally protects the entrapped drug from the external environment [41,42,43]. In addition, the photostability results were proportional to the LE results of the loading capacity experiment. This indicates that formulations with a high EE more effectively protect drugs from light.

### 3.5. ^1^O_2_ Photogeneration

**Figure 8:**
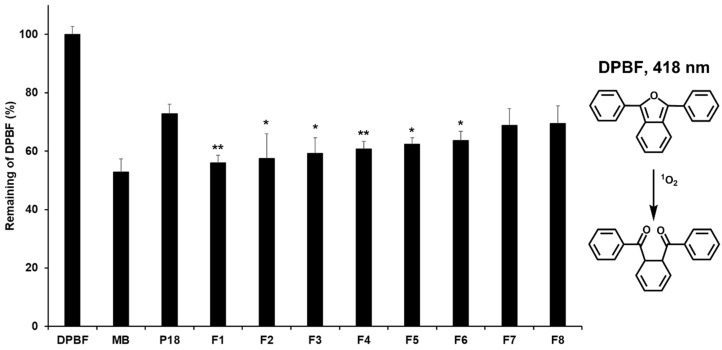
Study of DPBF (50 μM in DMSO) absorbance decay (%) for determining the ^1^O_2_ photogeneration efficacy of P18 with/without SLNs at 418 nm, after photoirradiation (total light dose, 2 J/cm^2^; irradiation time, 15 min). Results are expressed as means ± standard deviations of three independent experiments (*n* = 3). Negative control: DPBF (1,3-diphenylisobenzofuran); positive control: MB (methylene blue); P18 (purpurin-18). Statistical significance of the difference in DPBF absorbance between pure P18 solution and the formulations was determined using one-way analysis of variance and Student’s *t*-tests and is indicated by either a single asterisk (*p* < 0.05) or double asterisks (*p* < 0.01).

The pharmacological effects of P18 were first evaluated using the DPBF assay, which is a nonbiological assay. DPBF reacts with ^1^O_2_ generated after the photoirradiation of P18, which results in a decreased intensity of the DPBF absorption band. MB was used as a PC because it is a standard ^1^O_2_ sensitizer. The results indicated that the intensity of the DPBF absorption band after the irradiation of P18 with or without SLNs was higher than that after the irradiation of MB, which meant that the pharmacological effects of MB were higher than those of the test substances, as shown in Figure 8. The intensity values of the DPBF absorption band after the irradiation of pure P18 and the formulations were 72.75% and 55.96–69.57%, respectively. This indicates that P18 shows enhanced pharmacological effects when incorporated in the SLNs, meaning that SLNs can prevent the coalescence of P18 [28,39,40]. Interestingly, these results indicate that formulations with lower stability and lower LE had stronger pharmacological effects, suggesting that they have a high amount of nonentrapped drug with no coalescence, which results in a relatively high exposure to light and therefore more ^1^O_2_ generation [28,40]. However, considering the biological side-effects of the drug, it is important to express its pharmacological effects on target sites only. Therefore, this analysis was fruitful in terms of the selection of formulations based on the pharmacological effects, prior to the biological assay.

### 3.6. In Vitro Photoirradiation Studies

**Figure 9:**
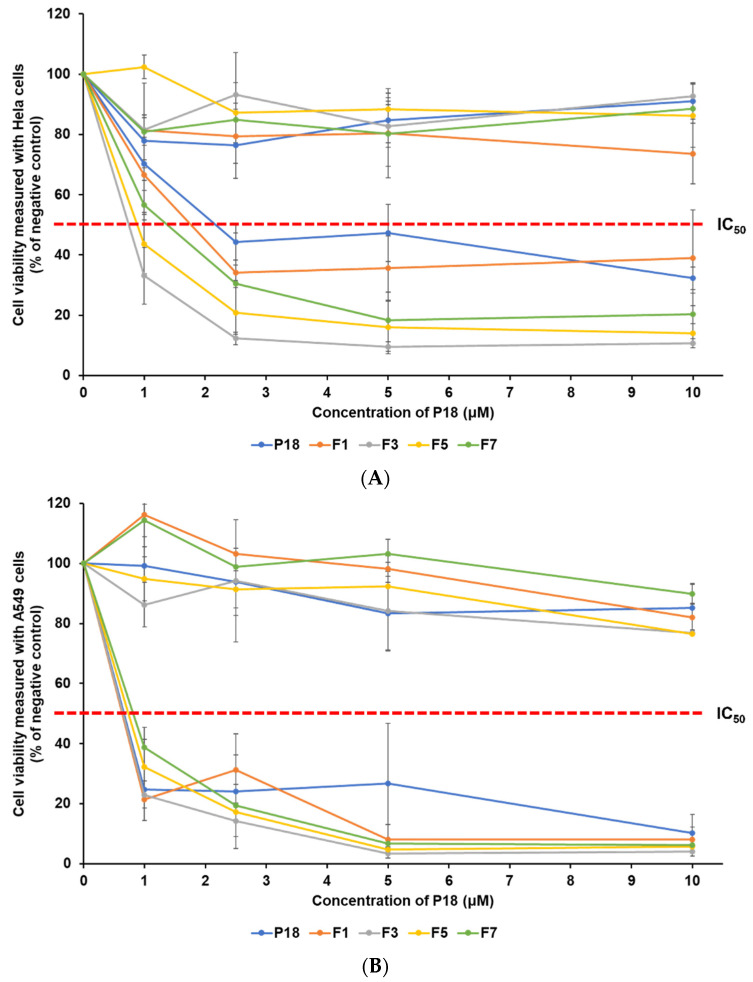
Viability of two cancer cell lines (HeLa and A549) treated with pure P18 solution, F1, F3, F5, and F7. The cell viability was measured using WST assay. (**A**) Dark and light cytotoxicity of HeLa cells and (**B**) dark and light cytotoxicity of A549 cells. Results are expressed as means ± standard deviations of three independent experiments (*n* = 3).

The photocytotoxic effects of P18 against human cervical carcinoma (HeLa) and human lung epithelial carcinoma (A549) were investigated. The viability of the cancer cells was estimated using the WST assay, as shown in Figure 9. The safety of the test substances for these cells was determined based on their viability under dark conditions. The viability of cancer cells under light conditions was evaluated to investigate the anticancer effects of P18-loaded SLNs. Various concentrations of each sample (1, 2.5, 5, and 10 μM) were used to estimate the inhibitory medium concentration values (IC_50_), as summarized in Table 5. Photo-cytotoxicity analysis was performed to evaluate the effect of different lipids (F1 (LA) and F3 (GMS)), as well as the concentration of GMS (F3, F5, and F7). The dark cytotoxicity analysis showed that all test substances were safe for both HeLa and A549 cells, ranging from 73.61% to 102.43% and 76.53% to 116.25%, respectively.

The analysis of viability of cancer cell lines after photoirradiation indicated that all formulations had an anticancer effect that was dependent on the concentration of P18, as shown in Figure 9. Upon comparing the IC_50_ values (μM), the order of PDT activity was F3 > F5 > F7 > F1 > P18 in HeLa cells, and F1 ≥ F3 ≥ P18 > F5 > F7 in A549 cells. This indicated that the effect of PDT in all formulations was cell type-dependent. In HeLa cells, all formulations tended to improve the anticancer effect compared to pure P18. However, in A549 cells, all formulations showed a PDT effect comparable to that of pure P18 (IC_50_ within 0.16 μM), even though F5 and F7 (IC_50_ 0.74 and 0.82 μM, respectively) showed slightly lower PDT effects than pure P18 did (0.66 μM). In both cell lines, the order of the PDT effect for all formulations was consistent with the order of decreasing particle size, except in the case of F1. Among all formulations, F3 containing GMS (lipid) and TW 80 (surfactant) exhibited the best PDT effect against both cell lines, using the smallest particle size to induce easy cellular uptake, followed by drug release and ^1^O_2_ photogeneration. Interestingly, F1 exhibited the best PDT effect against A549 cells, even though it had the largest particle size (ca. 763 nm, approximately four times that of F3) with the lowest EE (ca. 90%). This might be because of the lowest stability with a low zeta potential (−16.77 mV), which would maximize P18 release, leading to maximum singlet oxygen photogeneration. The difference in the lipid types in F1 and F3 resulted in a large difference in particle size, resulting in a relative difference in the PDT effect on HeLa cells. However, in A549 cells, a large difference in particle size did not cause a significant difference in the PDT effect. Generally, particle size and loading capacity are known to affect cellular uptake and expression of anticancer effects [1,21,22,34]. The differences in lipid concentration among F3, F5, and F7 caused relative differences in particle size, stability based on zeta potential, drug release, singlet oxygen photogeneration, and loading capacity, and the effect of particle size appears to be stronger than the effects of other parameters for anticancer activity.

### 3.7. Cellular Accumulation Study

**Figure 10:**
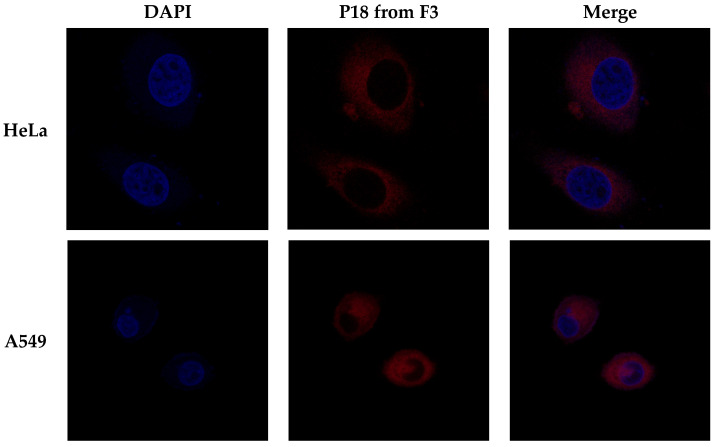
Confocal images of P18 cellular uptake for two cancer cell lines (HeLa and A549) following a 24 h exposure of F3. DAPI (diamidino-2-phenylindole): nuclear dye agent (blue); P18: purpurin-18 (red).

Confocal microscopy was used to determine the cellular uptake of P18 when administrating P18-loaded SLNs to tumor cells. Figure 10 shows the confocal images for two cancer cell lines (HeLa and A549) following a 24 h exposure of F3. F3, the highest PDT effect in the photocytotoxicity study, was subject to the cellular uptake study. The results demonstrated that P18 was highly accumulated in both cells but not nuclear without any toxicological effects causing cell apoptosis. This suggests that the SLN system could facilitate P18 uptake in cells, followed by drug release without any injury to cells leading to cell death. Thus, this result proved that both P18 and SLN were nonirritant for HeLa and A549 cells under the dark condition. In addition, P18 affected anti-cancer effects of PDT when irradiated by light, as mentioned in photocytotoxicity.

## 4. Conclusions

The purpose of this study was to synthesize and design P18- and P18-loaded SLNs, for innovative treatment of tumors using PDT. ^1^H-NMR analysis indicated that the six-membered purpurin ring was successfully formed in the synthesized P18. The FTIR analysis of P18-loaded SLNs confirmed that P18 was entrapped in lipids (LA, PA, and GMS) through both H-bonding and van der Waals forces. In particular, the formulations containing GMS had additional H-bonds when compared with those containing LA or PA, which resulted in a relatively small particle size. The drug release analysis indicated a burst release in formulations containing LA or PA and a sustained release in the formulations containing GMS. The photoirradiation analysis using two tumor cell lines (HeLa and A549) showed that the formulations were toxic under light conditions but not under dark conditions. In addition, the PDT effect of the formulations was cell type- as well as particle size-dependent. Among the formulations, F3 exhibited the best PDT effect against both cell lines because of the smallest particle size causing easy cellular uptake, followed by drug release and ^1^O_2_ photogeneration. Interestingly, the PDT effect of F1 was almost identical to that of F3 against A549 cells even though its particle size was about 4 times larger, which might be attributed to the highest drug release amount as well as ^1^O_2_ photogeneration among all formulations. These results might be observed because the particle size effect was the dominant factor determining the EPR effect. Therefore, the results of this study suggest that the P18-loaded SLNs are useful for PDT for anticancer treatment.

## Figures and Tables

**Table 1:** Compositions of P18-loaded SLNs.

Formulation	Drug (mg)	Lipid (mg)			Surfactant (mg)
	P18	LA	PA	GMS	TW 80
F1	10	100			200
F2	10		100		200
F3	10			100	200
F4	10			100	400
F5	10			300	200
F6	10			300	400
F7	10			500	200
F8	50			500	400

P18, purpurin-18; LA, lauric acid; PA, palmitic acid; GMS, glycerol monostearate; TW 80, Tween^®^ 80.

**Table 2:** Precision data obtained from the developed analytical method for P18.

No.	Recovery (%)
1	101.56
2	101.75
3	100.50
4	101.39
5	100.77
6	100.55
Average	101.09
SD	0.50
RSD (%)	0.49

**Table 3:** Accuracy data obtained from the developed analytical method for P18.

Drug (ppm)	No.	Recovery (%)	Average	SD	RSD (%)
1	1	97.13	99.25	1.51	1.52
	2	100.54			
	3	100.08			
5	1	99.42	100.16	0.52	0.52
	2	100.50			
	3	100.55			
20	1	100.12	100.27	0.14	0.14
	2	100.46			
	3	100.24			

**Table 4:** Infrared absorption (cm^−1^) of both P18 structural components (NH; C=O; and C=C stretching) and lipid structural components (OH; CH_2_, CH_3_; C=O (ester) stretching) in P18, lipids (LA, PA, and GMS), and formulations (F1, F2, and F3).

Test Substance	NH (cm^−1^)	C=O (cm^−1^)	C=C (cm^−1^)	OH (cm^−1^)	CH_2_, CH_3_ (cm^−1^)	C=O (Ester) (cm^−1^)
P18	3334	1714	1603	-	-	-
LA	-	1702	-	3322	2919, 2851	-
PA	-	1702	-	3322	2919, 2851	-
GMS	-	-	-	3244	2919, 2851	1734
F1	-	1638	-	3304	-	-
F2	-	1638	-	3304	-	-
F3	-	1638	-	3304	-	-

**Table 5:** IC_50_ (μM) values of HeLa and A549 cells; particle size and entrapment efficiency (EE) of pure P18 solution, F1, F3, F5, and F7.

Test Substance	Hela (μM)	A549 (μM)	Particle Size (nm)	EE (%)
P18	2.17	0.66	N/A	N/A
F1	1.77	0.64	762.53 ± 65.75	89.77 ± 3.61
F3	0.75	0.65	191.03 ± 1.80	90.20 ± 3.55
F5	0.89	0.74	243.67 ± 3.88	91.75 ± 3.23
F7	1.38	0.82	271.47 ± 2.90	93.10 ± 3.05

N/A, not applicable.

## Data Availability

The data presented in this study are available in [Synthesis and Design of Purpurin-18-Loaded Solid Lipid Nanoparticles for Improved Anticancer Efficiency of Photodynamic Therapy].

## References

[1] Kim J., Jo Y.-U., Na K. (2020). Photodynamic therapy with smart nanomedicine. Arch. Pharm. Res..

[2] Siwawannapong K., Zhang R., Lei H., Jin Q., Tang W., Dong Z., Lai R.-Y., Liu Z., Kamkaew A., Cheng L. (2020). Ultra-small pyropheophorbide-a nanodots for near-infrared fluorescence/photoacoustic imaging-guided photodynamic therapy. Theranostics.

[3] Oshiro-Junior J.A., Sato M.R., Boni F.I., Santos K.L.M., de Oliveira K.T., de Freitas L.M., Fontana C.R., Nicholas D., McHale A., Callan J.F. (2020). Phthalocyanine-loaded nanostructured lipid carriers functionalized with folic acid for photodynamic therapy. Mater. Sci. Eng. C.

[4] Wang Y.Y., Sun H., Guo D.S. (2019). Type I photodynamic therapy by organic–inorganic hybrid materials: From strategies to applications. Coord. Chem. Rev..

[5] Huang Y.Y., Balasubramanian T., Yang E., Luo D., Diers J.R., Bocian D.F., Lindsey J.S., Holten D., Hamblin M.R. (2012). Stable synthetic bacteriochlorins for photodynamic therapy: Role of dicyano peripheral groups, central metal substitution (2H, Zn, Pd), and Cremophor EL delivery. ChemMedChem.

[6] Tian J., Zhang W. (2019). Synthesis, self-assembly and applications of functional polymers based on porphyrins. Prog. Polym. Sci..

[7] Saga Y., Ishitani A., Takahashi N., Kawamura K. (2015). Production of bacteriopurpurin-18 phytyl ester from bacteriopheophytin a via allomerization by contact with titanium oxides in the presence of molecular oxygen. Bioorg. Med. Chem. Lett..

[8] Zhang Y., Zhang H., Wang Z., Jin Y. (2018). pH-Sensitive graphene oxide conjugate purpurin-18 methyl ester photosensitizer nanocomplex in photodynamic therapy. New J. Chem..

[9] Ranjitha S., Rajarajan G., Gnanendra T., Anbarasan P., Aroulmoji V. (2015). Structural and optical properties of Purpurin for dye-sensitized solar cells. Spectrochim. Acta A Mol. Biomol. Spectrosc..

[10] Gibbons D., Flanagan K.J., Pounot L., Senge M.O. (2019). Structure and conformation of photosynthetic pigments and related compounds. 15. Conformational analysis of chlorophyll derivatives–implications for hydroporphyrins in vivo. Photochem. Photobiol. Sci..

[11] Kimani S., Ghosh G., Ghogare A., Rudshteyn B., Bartusik D., Hasan T., Greer A. (2012). Synthesis and characterization of mono-, di-, and tri-poly (ethylene glycol) chlorin e6 conjugates for the photokilling of human ovarian cancer cells. J. Org. Chem..

[12] Rapozzi V., Zorzet S., Zacchigna M., Drioli S., Xodo L.E. (2013). The PDT activity of free and pegylated pheophorbide a against an amelanotic melanoma transplanted in C57/BL6 mice. Investig. New Drugs.

[13] Jeong Y.I., Yoo S.Y., Heo J., Kang D.H. (2019). Chlorin e6-Conjugated and PEGylated Immune Checkpoint Inhibitor Nanocomposites for Pulmonary Metastatic Colorectal Cancer. ACS Omega.

[14] Rapozzi V., Zacchigna M., Biffi S., Garrovo C., Cateni F., Stebel M., Zorzet S., Bonora G.M., Drioli S., Xodo L. (2010). Conjugated PDT drug: Photosensitizing activity and tissue distribution of PEGylated pheophorbide *a*. Cancer Biol. Ther..

[15] Srivatsan A., Ethirajan M., Pandey S.K., Dubey S., Zheng X., Liu T.-H., Shibata M., Missert J., Morgan J., Pandey R.K. (2011). Conjugation of cRGD peptide to chlorophyll a based photosensitizer (HPPH) alters its pharmacokinetics with enhanced tumor-imaging and photosensitizing (PDT) efficacy. Mol. Pharm..

[16] Thomas N., Bechet D., Becuwe P., Tirand L., Vanderesse R., Frochot C., Guillemin F., Barberi-Heyob M. (2009). Peptide-conjugated chlorin-type photosensitizer binds neuropilin-1 in vitro and in vivo. J. Photochem. Photobiol. B Biol..

[17] Yu L., Wang Q., Wong R.C.H., Zhao S., Ng D.K., Lo P.C. (2019). Synthesis and biological evaluation of phthalocyanine-peptide conjugate for EGFR-targeted photodynamic therapy and bioimaging. Dyes Pigm..

[18] Nguyen L., Li M., Woo S., You Y. (2019). Development of prodrugs for PDT-based combination therapy using a singlet-oxygen-sensitive linker and quantitative systems pharmacology. J. Clin. Med..

[19] Oluwole D.O., Manoto S.L., Malabi R., Maphanga C., Ombinda-Lemboumba S., Mthunzi-Kufa P., Nyokong T. (2018). Evaluation of the photophysicochemical properties and photodynamic therapy activity of nanoconjugates of zinc phthalocyanine linked to glutathione capped Au and Au_3_Ag_1_ nanoparticles. Dyes Pigment..

[20] Kwon J.-G., Song I.-S., Kim M.-S., Lee B.H., Kim J.H., Yoon I., Shim Y.K., Kim N., Han J., Youm J.B. (2013). Pu-18-*N*-butylimide-NMGA-GNP conjugate is effective against hepatocellular carcinoma. Integr. Med. Res..

[21] Li Z., Huang J., Wu J. (2021). pH-Sensitive nanogels for drug delivery in cancer therapy. Biomater. Sci..

[22] Bouramtane S., Bretin L., Pinon A., Leger D., Liagre B., Perez D.D.S., Launay Y., Brégier F., Sol V., Chaleix V. (2021). Acetylxylan-pheophorbide-a nanoparticles designed for tumor-targeted photodynamic therapy. J. Appl. Polym. Sci..

[23] Gu W., Meng F., Haag R., Zhong Z. (2021). Actively targeted nanomedicines for precision cancer therapy: Concept, construction, challenges and clinical translation. J. Control. Release.

[24] Kamal M.M., Salawi A., Lam M., Nokhodchi A., Abu-Fayyad A., El Sayed K.A., Nazzal S. (2020). Development and characterization of curcumin-loaded solid self-emulsifying drug delivery system (SEDDS) by spray drying using Soluplus^®^ as solid carrier. Powder Technol..

[25] Chen J., Lu W.-L., Gu W., Lu S.-S., Chen Z.-P., Cai B.-C., Yang X.-X. (2014). Drug-in-cyclodextrin-in-liposomes: A promising delivery system for hydrophobic drugs. Expert Opin. Drug Deliv..

[26] Makwana V., Jain R., Patel K., Nivsarkar M., Joshi A. (2015). Solid lipid nanoparticles (SLN) of Efavirenz as lymph targeting drug delivery system: Elucidation of mechanism of uptake using chylomicron flow blocking approach. Int. J. Pharm..

[27] Yasir M., Gaur P.K., Puri D., Shehkar P., Kumar S.S. (2018). Solid lipid nanoparticles approach for lymphatic targeting through intraduodenal delivery of quetiapine fumarate. Curr. Drug Deliv..

[28] Zoubari G., Staufenbiel S., Volz P., Alexiev U., Bodmeier R. (2017). Effect of drug solubility and lipid carrier on drug release from lipid nanoparticles for dermal delivery. Eur. J. Pharm. Biopharm..

[29] Peng T.-X., Liang D.-S., Guo F., Peng H., Xu Y.-C., Luo N.-P., Zhang X.-Y., Zhong H.-J. (2019). Enhanced storage stability of solid lipid nanoparticles by surface modification of comb-shaped amphiphilic inulin derivatives. Colloids Surf. B Biointerfaces.

[30] Gupta T., Singh J., Kaur S., Sandhu S., Singh G., Kaur I.P. (2020). Enhancing bioavailability and stability of curcumin using solid lipid nanoparticles (CLEN): A covenant for its effectiveness. Front. Bioeng. Biotechnol..

[31] Duan Y., Dhar A., Patel C., Khimani M., Neogi S., Sharma P., Kumar N.S., Vekariya R.L. (2020). A brief review on solid lipid nanoparticles: Part and parcel of contemporary drug delivery systems. RSC Adv..

[32] Han L., Wang T. (2016). Preparation of glycerol monostearate from glycerol carbonate and stearic acid. RSC Adv..

[33] Smith K.M., Goff D.A. (1985). Synthesis of nickel (II) isobacteriochlorins from nickel (II) complexes of chlorophyll derivatives. J. Am. Chem. Soc..

[34] Lima A.M., Dal Pizzol C., Monteiro F.B., Creczynski-Pasa T.B., Andrade G.P., Ribeiro A.O., Perussi J.R. (2013). Hypericin encapsulated in solid lipid nanoparticles: Phototoxicity and photodynamic efficiency. J. Photochem. Photobiol. B Biol..

[35] Alépée N., Tornier C., Robert C., Amsellem C., Roux M.-H., Doucet O., Pachot J., Méloni M., de Fraissinette A.d.B. (2010). A catch-up validation study on reconstructed human epidermis (SkinEthic™ RHE) for full replacement of the Draize skin irritation test. Toxicol. In Vitro.

[36] Yeo S., Yoon I., Lee W.K. (2022). Design and Characterisation of pH-Responsive Photosensitiser-Loaded Nano-Transfersomes for Enhanced Photodynamic Therapy. Pharmaceutics.

[37] Zacharis C.K., Vastardi E. (2018). Application of analytical quality by design principles for the determination of alkyl p-toluenesulfonates impurities in aprepitant by HPLC. Validation using total-error concept. J. Pharm. Biomed..

[38] Hassanpour P., Hamishehkar H., Baradaran B., Mohammadi M., Shomali N., Spotin A., Hazratian T., Nami S. (2020). An appraisal of antifungal impacts of nano-liposome containing voriconazole on voriconazole-resistant Aspergillus flavus isolates as a groundbreaking drug delivery system. Nanomed. Res. J..

[39] Khatak S., Semwal U.P., Pandey M.K., Dureja H. (2018). Investigation of Antimycobacterial potential of solid lipid nanoparticles against M. smegmatis. J. Pharm. Innov..

[40] Vivek K., Reddy H., Murthy R.S. (2007). Investigations of the effect of the lipid matrix on drug entrapment, in vitro release, and physical stability of olanzapine-loaded solid lipid nanoparticles. AAPS Pharmscitech..

[41] Radomska-Soukharev A. (2007). Stability of lipid excipients in solid lipid nanoparticles. Adv. Drug Deliv. Rev..

[42] Kuklenyik Z., Jones J.I., Gardner M.S., Schieltz D.M., Parks B.A., Toth C.A., Rees J.C., Andrews M.L., Carter K., Lehtikoski A.K. (2018). Core lipid, surface lipid and apolipoprotein composition analysis of lipoprotein particles as a function of particle size in one workflow integrating asymmetric flow field-flow fractionation and liquid chromatography-tandem mass spectrometry. PLoS ONE.

[43] Dodangeh M., Tang R.-C., Gharanjig K. (2019). Improving the photostability of curcumin using functional star-shaped polyamidoamine dendrimer: Application on PET. Mater. Today Commun..

